# Ein Clip zu viel

**DOI:** 10.1007/s00120-022-02013-0

**Published:** 2023-01-06

**Authors:** Marlene Felsner, Bernhard Grubmüller

**Affiliations:** 1 Klinische Abteilung für Urologie, Universitätsklinikum Krems, Mitterweg 10, 3500 Krems, Österreich; 2 Karl Landsteiner Privatuniversität für Gesundheitswissenschaften, Dr. Karl-Dorrek-Straße 30, 3500 Krems, Österreich

**Keywords:** Harnblasenkarzinom, Hydronephrose, Koloskopie, Urologie, Mainz-II-Pouch, Bladder cancer, Hydronephrosis, Colonoscopy, Urology, Mainz II pouch

## Abstract

Ein Patient mit Mainz-II-Pouch aufgrund eines muskelinvasiven Harnblasenkarzinoms wurde nach Nachsorgekolonoskopie mit De-novo-Hydronephrose links vorstellig. Ursächlich hierfür war eine Abtragung und Clippung der Neoostien als Polypen bei der Kolonoskopie. In einem Rendez-vous-Verfahren aus antegrader Ureterenoskopie und Kolonoskopie nach Nephrostomieanlage links konnte ein Gallengangstent zur Dilatation in das linke Neostium einlegt werden und der Harnabfluss wieder hergestellt werden, welcher nach Stentabgang permanent nachzuweisen war.

## Abstract (en)

A patient with a Mainz II pouch due to muscle invasive bladder cancer presented to our urology department with de novo hydronephrosis on the left kidney after a regular colonoscopy. In the course of diagnostic workup, it was found that the neo-ostia were removed and clipped as a “polyp”. Via rendezvous technique of antegrade ureterenoscopy and colonoscopy after nephrostomy placement urine outflow was successfully reconstituted by inserting a biliary stent into the left neo-ostium, which consolidated after stent removal as a permanent state.

## Anamnese

Ein der Abteilung bekannter 74-jähriger Patient wurde in der Notaufnahme vorstellig wegen Bauchschmerzen mit punctum maximum im linken Unterbauch nach Kolonoskopie vor 4 Tagen im Rahmen der Nachsorge eines Mainz-Pouch II (Ureterosigmoideostomie). Es seien im Rahmen der Kolonoskopie im niedergelassenen Bereich zwei Polypen problemlos abgetragen worden.

Die Erstdiagnose eines muskelinvasiven Urothelkarzinoms des Blasenhalses und der prostatischen Harnröhre erhielt der Patient vor 4 Jahren. Es erfolgte 4 Wochen nach Diagnosestellung bei negativem Staging mittels Computertomographie (CT) von Thorax und Abdomen die primäre radikale Zystoprostatektomie mit Mainz-Pouch II, auf Patientenwunsch ohne neoadjuvante Chemotherapie (nach ausführlicher Aufklärung über Vorteile/Nachteile/Risiken/Komplikationen der neoadjuvanten systemischen Therapie). Da der Patient eine kontinente Harnableitung forcierte und sich auch der initiale intraoperative Schnellschnitt des Harnröhrenstumpfs positiv zeigte (weshalb hier der externe urethrale Spinkter zum großen Teil reseziert werden musste), fiel die definitive Entscheidung zur Harnableitung auf eine Ureterosigmoideostomie.

## Befund

In der Bed-side-Sonographie durch den diensthabenden Urologen zeigte sich eine De-novo-Hydronephrose Grad III links und Grad I rechts, die Blutgasanalyse war ausgeglichen. Im Labor wurde eine CRP-Erhöhung von 9 mg/dl bei Leukozyten im Normbereich (7 G/l) und Kreatinin von 1 mg/dl festgestellt. Das Abdomen nativ-Röntgen zeigte eine Kolonblähung ohne freie Luft oder Ileus sowie einen metalldichten Clip im linken Unterbauch.

## Diagnostik

**Figure 1:**
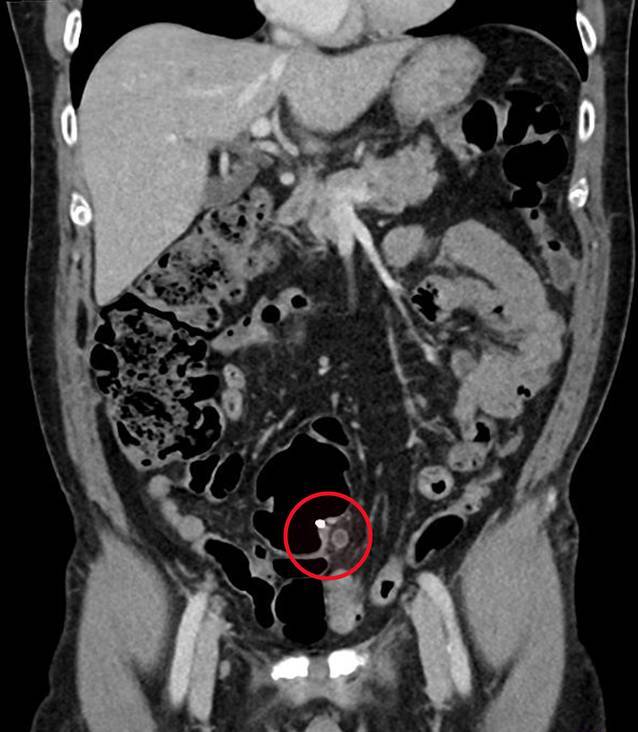

**Figure 2:**
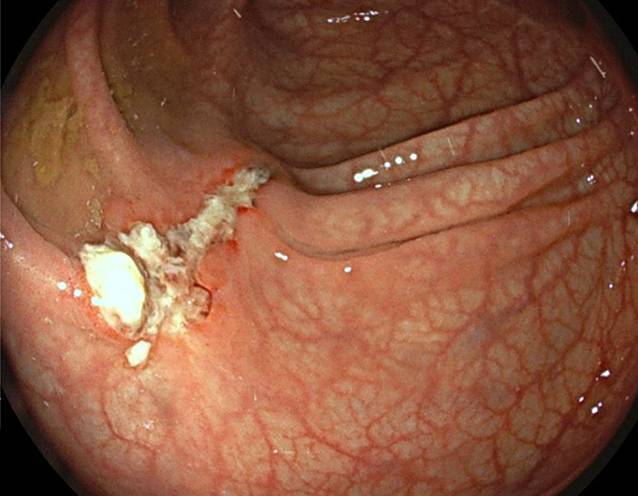

Nach stationärer Aufnahme zur weiteren Abklärung wurde eine Kontrastmittel-CT des Abdomens durchgeführt, welche die Hydronephrose Grad III links auf eine Obstruktion durch den Endoskopieclip zurückführte (Abb. 1). In einer Akutkolonoskopie wurde der Mainz-Pouch II als normal befundet, jedoch konnten die Ureterenmündungen beidseits – bis dato in den jährlichen Nachsorgekolonoskopien im intramuralen Bereich unauffällig – nicht dargestellt werden. Es zeigten sich lediglich zwei fibrinbedeckte Polypenabtragungsstellen (Abb. 2). In Zusammenschau der Befunde waren die Ureterenmündungen im Pouch als vermeintliche Polypen abgetragen, verödet und geclippt worden. Der in der CT beschriebene Clip war zwischenzeitlich offenbar abgegangen.

## Therapie und Verlauf

Bei einem CRP-Anstieg unter antibiotischer Therapie auf 18 mg/dl wurde der Patient am nächsten Tag an der linken Niere mit einer perkutanen Nephrostomie versorgt. Es zeigte sich antegrad ein kompletter Kontrastmittelstopp vor der Einmündung in den Pouch.

Der zwischenzeitlich eingetroffene, histologische Befund der „Polypen“ zeigte einen unauffälligen Befund. Eine Harnleiterneuimplantation in den Pouch wurde mit dem Patienten diskutiert, von diesem jedoch abgelehnt.

**Figure 3:**
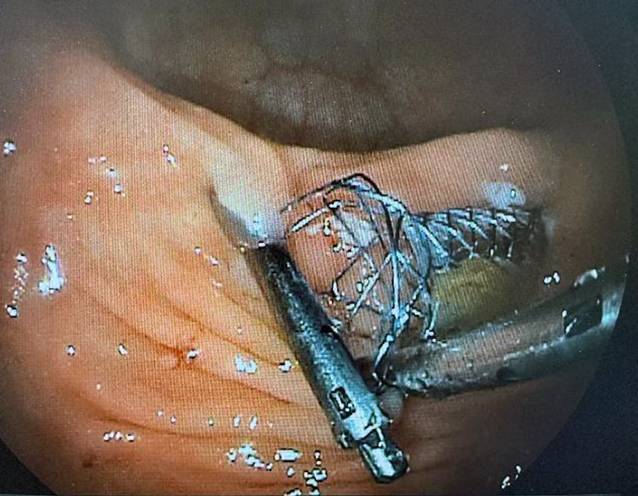

Im weiteren Verlauf wurde der Patient geplant zum Rendez-vous-Verfahren aus Kolonoskopie und antegrader Ureterorenoskopie für ein endoskopisches Realignment aufgenommen, wo es gelang, nach Vorlegen eines Führungsdrahtes einen Gallengangstent in die Ureterenmündung zur kontinuierlichen Dilatation einzubringen (Abb. 3). Unter antibiotischer Abschirmung wurde die perkutane Nephrostomie geklemmt. Der Gallengangstent wurde vom Patienten nach 4 Wochen ausgeschieden, sonographisch zeigte sich weiterhin eine Hydronephrose Grad I links bei unauffälligen Nierenfunktionsparametern.

**Figure 4:**
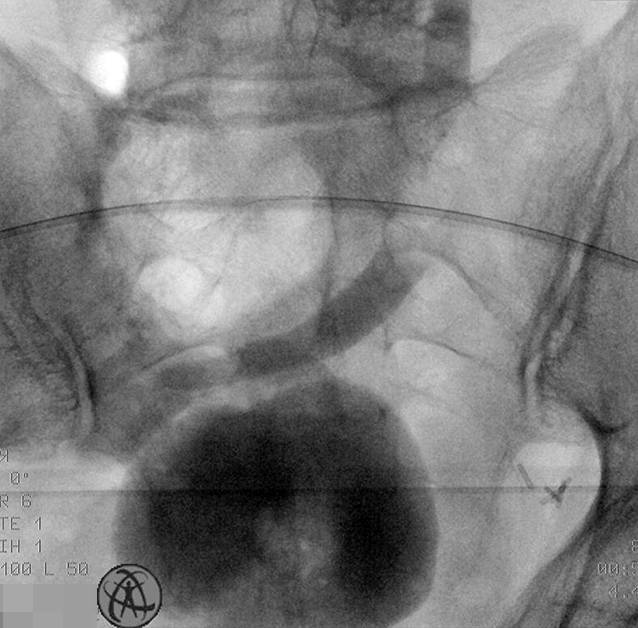

In nachfolgenden Kontrollen per Röntgen-gestützter, antegrader Ureteropyelographie über die liegende perkutane Nephrostomie konnte ein steter Abfluss über die Ureterenmündung in den Pouch dargestellt werden (Abb. 4). Bei klinischer Beschwerdefreiheit wurde die liegende perkutane Nephrostomie 4 Wochen nach Stentverlust entfernt.

Der Patient findet sich derzeit in regelmäßiger Nachsorge mit sonographischen Verlaufskontrollen, Blutgasanalysen und Laborkontrollen.

## Diskussion

Der Patient wurde lange Zeit im intramuralen Bereich per Kolonoskopie komplikationslos nachgesorgt. Auch die vorletzte Kolonoskopie im extramuralen Bereich sei unauffällig verlaufen. Zuletzt war es jedoch zu einem Untersucherwechsel und damit einhergehend zu einem Informationsverlust gekommen, weshalb die Beurteilung des Mainz-Pouch II mit seinen anatomischen Gegebenheiten offensichtlich nicht suffizient durchgeführt werden konnte. Essentiell hierbei könnten die Dokumentation mittels Bildmaterials im Rahmen der Kolonoskopie und auch die konsequente Dokumentation der beim Mainz-Pouch II typischen Veränderungen sein, die möglicherweise in der betreuenden Klinik besser vorliegen.

Der beschriebene Therapiealgorithmus zog einige Eingriffe in Narkose mit sich. Von Anfang an diskutiert wurde eine Ureterneuimplantation in den Mainz-Pouch II. Dieser wurde vom Patienten aber mehrfach abgelehnt. Sollte es zu vermehrten Infekten und einer Rezidivstenose kommen, könnte ein solcher Eingriff jedoch unumgänglich werden. Alternativ sollte über regelmäßige Neoostiumdilatationen nachgedacht werden, da die Einmündungsstelle im Verlauf narbigen Veränderungen entwickeln können.

## Fazit für die Praxis

- Die Anbindung der Nachsorgekolonoskopien nach Ureterosigmoideostomien an den intramuralen Bereich kann zur Vermeidung von Informationsverlust und damit einhergehenden iatrogenen Verletzungen beitragen.
- Der Fallbericht verdeutlicht die Bedeutung der interdisziplinären Zusammenarbeit in der onkologischen und chirurgischen Nachsorge.

